# Pyruvate kinase M2 regulates kidney fibrosis through pericyte glycolysis during the progression from acute kidney injury to chronic kidney disease

**DOI:** 10.1111/cpr.13548

**Published:** 2023-09-25

**Authors:** Yulan Chen, Xueyuan Bai, Jianwen Chen, Mengjie Huang, Quan Hong, Qing Ouyang, Xuefeng Sun, Yan Zhang, Jiaona Liu, Xu Wang, Lingling Wu, Xiangmei Chen

**Affiliations:** ^1^ Department of Nephrology First Medical Center of Chinese PLA General Hospital, National Key Laboratory of Kidney Diseases, National Clinical Research Center for Kidney Diseases, Beijing Key Laboratory of Kidney Diseases Research Beijing China; ^2^ Chinese PLA Medical School Beijing China

## Abstract

We aimed to investigate the role of renal pericyte pyruvate kinase M2 (PKM2) in the progression of acute kidney injury (AKI) to chronic kidney disease (CKD). The role of PKM2 in renal pericyte‐myofibroblast transdifferentiation was investigated in an AKI‐CKD mouse model. Platelet growth factor receptor beta (PDGFRβ)‐iCreERT2; tdTomato mice were used for renal pericyte tracing. Western blotting and immunofluorescence staining were used to examine protein expression. An 5‐ethynyl‐2′‐deoxyuridine assay was used to measure renal pericyte proliferation. A scratch cell migration assay was used to analyse cell migration. Seahorse experiments were used to examine glycolytic rates. Enzyme‐linked immunoassay was used to measure pyruvate kinase enzymatic activity and lactate concentrations. The PKM2 nuclear translocation inhibitors Shikonin and TEPP‐46 were used to alter pericyte transdifferentiation. In AKI‐CKD, renal pericytes proliferated and transdifferentiated into myofibroblasts and PKM2 is highly expressed in renal pericytes. Shikonin and TEPP‐46 inhibited pericyte proliferation, migration, and pericyte‐myofibroblast transdifferentiation by reducing nuclear PKM2 entry. In the nucleus, PKM2 promoted downstream lactate dehydrogenase A (LDHA) and glucose transporter 1 (GLUT1) transcription, which are critical for glycolysis. Therefore, PKM2 regulates pericyte glycolytic and lactate production, which regulates renal pericyte‐myofibroblast transdifferentiation. PKM2‐regulated renal pericyte‐myofibroblast transdifferentiation by regulating downstream LDHA and GLUT1 transcription and lactate production. Reducing nuclear PKM2 import can reduce renal pericytes‐myofibroblasts transdifferentiation, providing new ideas for AKI‐CKD treatment.

## Graphical abstract

Schematic diagram depicting proposed mechanisms of PKM2‐induced renal pericyte‐myofibroblast transdifferentiation through regulation of transcription levels of downstream LDHA and GLUT1 and lactate production. GLUT1, glucose transporter 1; LDHA, lactate dehydrogenase A; PKM2, pyruvate kinase M2; TGFβ1, transforming growth factor β1.

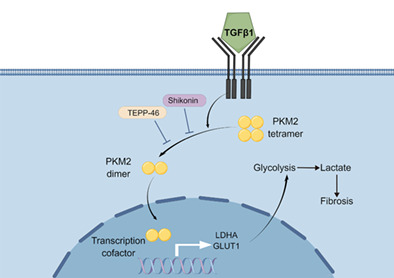

## 1 INTRODUCTION

Acute kidney injury (AKI) refers to a clinical syndrome in which renal excretory function declines sharply in a short period due to various aetiologies, ultimately leading to changes in renal structure and function.^1^ A meta‐analysis of 82 studies showed that the risk of chronic kidney disease (CKD) in AKI patients increased by 2.67 times, the risk of end‐stage renal disease increased by 4.81 times, and the risk of death increased by 1.80 times.^2^ Renal interstitial fibrosis is a key feature of AKI‐to‐CKD progression, and the secretion of extracellular matrix components by myofibroblasts plays an important role in renal interstitial fibrosis.^3^ Genetic fate tracing studies have shown that renal pericytes in the renal interstitium are a major source of myofibroblasts during fibrotic nephropathy.^4^, ^5^ However, the characteristics of pericyte fate and transition during AKI‐CKD still need to be further elucidated.

Pyruvate kinase (PK) is the key enzyme in glycolysis and catalyses the conversion of phosphoenolpyruvate to pyruvate.^6^ There are four subtypes of PK, among which pyruvate kinase M2 (PKM2) is mainly present in proliferating cells and plays a decisive role in metabolic reprogramming.^7^ Studies have shown high PKM2 expression in the renal interstitium in AKI‐induced CKD kidneys.^8^ Furthermore, PKM2 may be a specific biomarker for the early diagnosis of AKI.^9^ However, whether PKM2 is expressed in pericytes and whether it plays a role in pericyte fate transition in AKI‐CKD are still unclear.

In our study, an AKI‐CKD animal model was constructed, and the characteristics of pericytes were examined. Transgenic pericyte tracer mice were used to study pericyte fate transition and PKM2 expression in pericytes during AKI‐CKD. We established a pericyte‐myofibroblast transdifferentiation model in vitro and treated model cells with Shikonin and TEPP‐46 to examine the role and mechanism of PKM2 in this process.

## 2 MATERIALS AND METHODS

### 2.1 Animals

All animal protocols of our study were approved by the Institutional Animal Care and Use Committee of the PLA General Hospital and Military Medical College. Wild‐type C57BL/6J mice (male, aged 7 weeks) were acquired from the Jin Mu Yang Laboratory Animal Company (Beijing, China). All mice were kept in an environment with constant temperature (22°C) and humidity (70%) and alternating day and night cycles.

### 2.2 Establishment of the mouse AKI‐CKD model

For the AKI‐CKD model, the mice (male, aged 8–10 weeks, 20–25 g) were subjected to unilateral renal ischaemia–reperfusion injury (uIRI) in the left kidney. The mice were anaesthetized with pentobarbital sodium (70 mg/kg) and were placed on a heated surgical table to maintain a body temperature of 37°C. Unilateral renal ischaemia was induced by clamping the left renal pedicles for 40 min as previously described.^10^ The sham group underwent only a left flank incision without clamping of the blood vessels. The mice were allowed to recover for 24 h to 28 days after the clamps were removed. Contralateral nephrectomy was removed 24 h before sacrifice, at which time the mice were euthanized, and kidney tissue and serum were harvested.

Unilateral ureteral obstruction (UUO) model was generated by ligation of the left ureter ^11^ and kidney tissue was harvested for analysis.

To examine the role of PKM2 in AKI‐CKD, male mice were randomly assigned to four groups: the control‐vehicle group (*n* = 4), the control‐Shikonin/TEPP‐46 group (*n* = 4), the IRI‐vehicle group (*n* = 6) and the IRI‐Shikonin/TEPP‐46 group (*n* = 6). All mice underwent unilateral renal ischaemia (40 min) and reperfusion surgery as described previously. Mice were treated with the target reagent (1 mg/kg Shikonin or 30 mg/kg TEPP‐46 every other day) or vehicle (5% Dimethyl Sulfoxide ‘DMSO’ in saline) for 14 days. On the 14th day after reperfusion, blood and kidney samples were harvested for analysis.

### 2.3 Generation of pericyte‐labelled mice

PDGFRβ‐iCreERT2 mice (BIOCYTOGEN Company, cat. T110129) and tdTomato mice (GemPharmatech Company, cat. T002249) were mated to generate PDGFRβ‐iCreERT2; tdTomato mice. PDGFRβ‐iCreERT2; tdTomato mice carry the PDGFRβ promoter‐driven tamoxifen‐inducible Cre recombinase and the reporter LoxP‐STOP‐loxP‐tdTomato. Seven‐week‐old mice were intraperitoneally injected with tamoxifen (2 mg for 3 consecutive days) to induce Cre‐mediated recombination of LoxP sites and deletion of the STOP signal. As a result, red fluorescent protein (tdTomato) was expressed and labelled PDGFRβ^+^ cells and their progeny. The mice were free of tamoxifen for 3 weeks before the establishment of the IRI model. Mouse genotyping was performed by a Quick Genotyping Assay Kit for Mouse Tails (Beyotime, cat. D7283).

PDGFRβ‐iCreERT2; tdTomato mice were surgically implanted with abdominal imaging windows on the left side of the abdomen for intravital microscopy.^12^ The uIRI model was established at approximately 10 weeks. The kidney was observed with a two‐photon microscope in vivo.

PDGFRβ‐iCreERT2; mTmG mice were generated by PDGFRβ‐iCreERT2 mice and mTmG mice (BIOCYTOGEN Company, cat. T007575). PDGFRβ‐iCreERT2; mTmG mice expressed green fluorescent protein (GFP) in PDGFRβ^+^ cells upon tamoxifen induction. We established the IRI model in these mice and used GFP antibody for immunofluorescence staining to detect the localization of PDGFRβ.

### 2.4 Assessment of kidney injury

Serum creatinine (Scr) and blood urea nitrogen (BUN) levels were measured to assess kidney injury. The serum was separated by centrifugation at 3500 rpm for 15 min after the blood samples were collected from the vena cava at the indicated times. The serum was sent to the Biochemistry Department of PLA General Hospital for Scr and BUN level analysis.

### 2.5 Histological and immunohistochemical analysis

A quarter of the mouse kidney tissue was fixed in 10% formalin, dehydrated with an ethanol gradient and embedded in paraffin. Tissue sections (4 μm) were then subjected to Masson staining or immunohistochemical staining. For immunohistochemical staining, the samples were stained with antibodies against platelet‐derived growth factor receptor β (PDGFRβ, 1:100, Abcam, cat. ab32570), alpha smooth muscle actin (α‐SMA, 1:150, Abcam, cat. ab7817), GFP (1:500, Abcam, cat. ab6673), and PKM2 (1:100, CST, cat. 4053S) overnight, and the subsequent steps were performed according to the instructions of the immunohistochemical staining kit (ZSGB‐BIO).

### 2.6 Western blotting

Renal tissue or cells were lysed in radioimmunoprecipitation buffer (Beyotime) containing 1% phenylmethylsulfonyl fluoride (Solarbio) and 1% phosphorylase inhibitors (Psatong). The protein concentration of each sample was determined by a bicinchoninic acid assay (BCA) protein assay kit (Thermo Fisher Scientific). The samples were separated by 10% sodium dodecyl sulfate‐polyacrylamide gel electrophoresis (SDS‐PAGE) gels, and transferred to the membranes. The membranes were then blocked and incubated overnight at 4°C with primary antibodies. Then the membranes were washed with Tris‐buffered saline‐Tween 20 and incubated with secondary antibodies. The protein bands were visualized by chemiluminescence, and blot analysis was performed with ImageJ. All experiments were repeated three times. The primary antibodies used were as follows: proliferating cell nuclear antigen (PCNA, 1:1000, Abcam, ab92552), PDGFRβ (1:1000), platelet‐derived growth factor receptor α (PDGFRα, 1:1000, Abcam, cat. ab221154), α‐SMA (1:3000), β‐actin (1:10,000, Proteintech, cat. 66,009‐1‐1 g), glyceraldehyde‐3‐phosphate dehydrogenase (GAPDH; 1:10,000, Proteintech, 66004‐1‐1 g), PKM2 (1:1000), TATA binding protein (1:1000, Abcam, cat. ab818), p‐PKM2 Y105 (1:1000, CST, cat. 3827S), lactate dehydrogenase A (LDHA, 1:1000, CST, cat. 3582), and glucose transporter 1 (GLUT1, 1:200, Abcam, cat. ab150299).

Cell nuclei were extracted using a nuclear protein extraction kit (X‐Y Biotechnology, cat. XY91218).

### 2.7 Disuccinimidyl suberate crosslinking

Disuccinimidyl suberate (DSS)‐crosslinked pericytes were collected in lysis buffer. DSS (5 mM, Thermo Fisher Scientific, cat. 21655) was added and incubated at room temperature for 30 min to crosslink the cells, and the reaction was stopped with 10 mM Tris for 15 min. The samples were analysed by Western blotting.

### 2.8 Kidney pericyte isolation, culture, and treatment

Primary PDGFRβ‐positive (PDGFRβ^+^) cells were extracted from the kidneys of C57BL/6J mice and purified by magnetic bead cell sorting.^13^ The kidney cell suspensions were sequentially labelled with PDGFRβ‐PE antibodies (Miltenyi Biotec, cat. 130‐109‐745) and anti‐PE microbeads (Miltenyi Biotec, cat. 130‐048‐801). Pericytes were cultured in DMEM/F12 medium (Gibco, cat. 11330032) containing 10% foetal bovine serum. Half of the medium was changed every 2 days. Primary PDGFRβ^+^ cells underwent 1 passage before use. The cells were synchronized by serum starvation for 12 h. Then, to examine the effect of PKM2 on pericytes, PDGFRβ^+^ cells were treated with (1) vehicle (1‰ DMSO), (2) 5 ng/mL TGFβ1 (PeproTech, cat. 100–21), (3) TGFβ1 + 1 mM Shikonin (Aladdin‐e, S170939), or (4) TGFβ1 + 50 μM TEPP‐46 (Selleck, cat. S7302) for 48 h. Additionally, to examine the effect of lactate on pericyte‐myofibroblast transdifferentiation, pericytes were treated with or without 5 mM lactate in complete medium for 48 h.

### 2.9 Immunofluorescence staining of renal pericytes

Pericytes were cultured in chambers, fixed with 4% paraformaldehyde in phosphate buffer solution (PBS) for 30 min and permeabilized with 0.5% Triton for 15 min. The fixed cells were incubated overnight at 4°C with primary antibodies against α‐SMA (1:300), fibronectin (FN, 1:100, Abcam, cat. ab2413), and PKM2 (1:100). After being washed with PBS, the pericytes were incubated with secondary antibodies for 2 h at room temperature. The nuclei were counterstained with 4′,6‐diamidino‐2‐phenylindole (DAPI). Afterward, the sections were observed under a fluorescence microscope.

### 2.10 5‐Ethynyl‐2′‐deoxyuridine staining

Click‐iT® Plus 5‐ethynyl‐2′‐deoxyuridine (EdU) Imaging Kits (Thermo Fisher Scientific, cat. C10638) were used to analyse pericyte proliferation according to the manufacturer's instructions. DAPI was used to stain the nuclei.

### 2.11 Scratch cell migration assay

To study the effect of TEPP‐46 and Shikonin on pericyte migration, pericytes were scratched with 1000 mL pipette tips after synchronization. After being scratched, the pericytes were imaged and treated with TGFβ1 with/without Shikonin or TEPP‐46 for 48 h. After the treatments, images were obtained, saved, and analysed.

### 2.12 Seahorse experiments

Reagents for Seahorse glycolytic rate tests were purchased from Agilent. Seahorse experiments were performed according to the manufacturer's instructions on a Seahorse XFe 24 Analyser.

### 2.13 Enzyme‐linked immunoassay

Lactate levels in pericyte culture medium were detected using a lactate colorimetric/fluorometric assay kit (Solarbio, cat. BC2230) according to the manufacturer's protocol.

PK enzymatic activity in pericytes was detected using a PK activity assay kit (Sigma, cat. MAK072) according to the manufacturer's protocol.

### 2.14 Statistical analysis

GraphPad Prism (version 9.0) was used for data analysis and graph preparation. The data are expressed as the means ± SDs. Comparisons among groups were conducted using t tests or nonparametric tests. All *p*‐values were two‐sided, and *p* < 0.05 was considered significant.

## 3 RESULTS

### 3.1 Kidney fibrosis progressed, and renal pericytes proliferated in the IRI‐induced AKI‐CKD model

**FIGURE 1:**
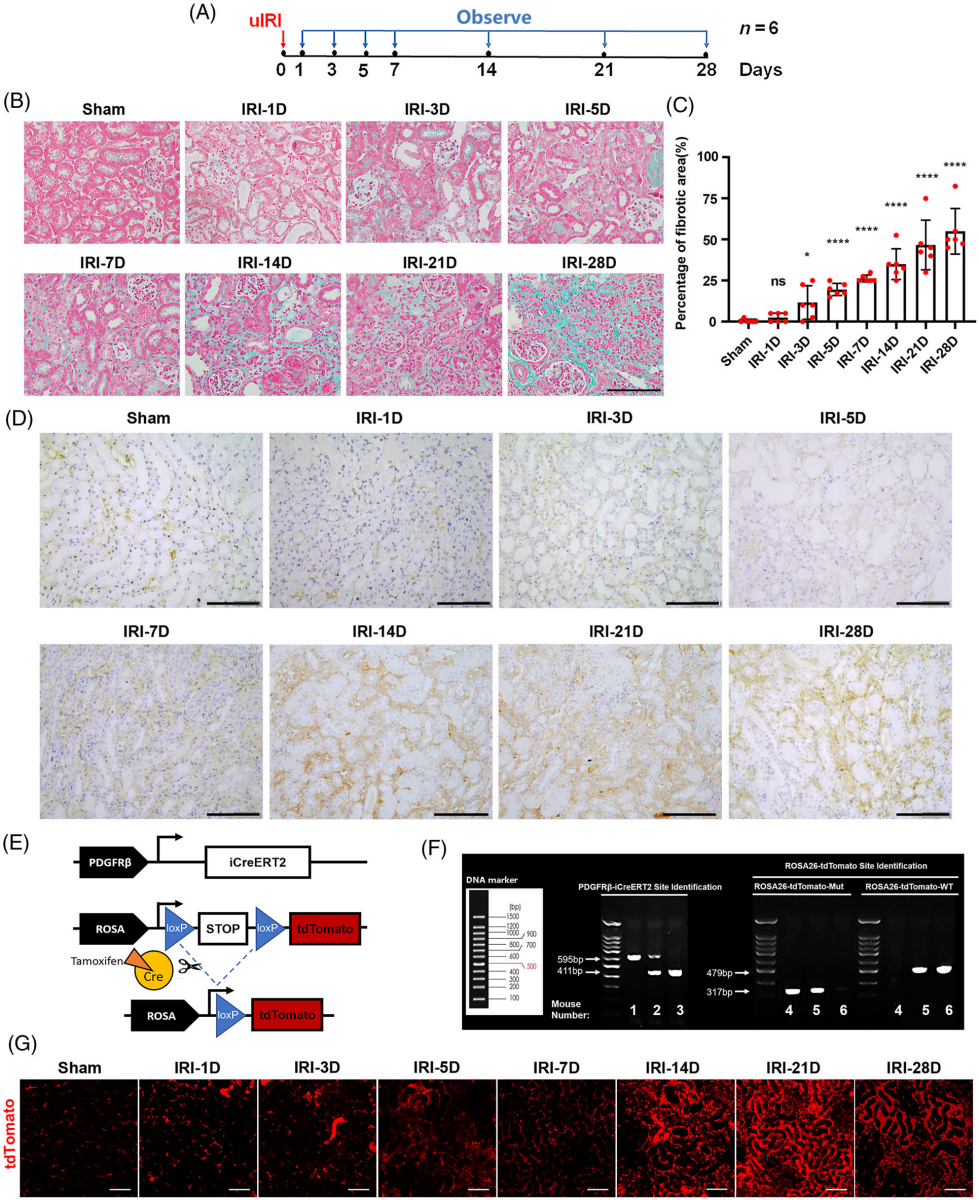
Pericytes increased in IRI‐associated renal interstitial fibrosis. (A) Experimental design. The red arrow indicated the establishment of renal ischaemia, and the blue arrows indicated the time of kidney reperfusion and animal sacrifice. (B,C) Masson staining of kidney tissue and the percentage of the fibrotic area (*n* = 6). (D) Location of platelet growth factor receptor beta (PDGFRβ) in mouse IRI kidney samples, as investigated by immunohistochemical staining. (E) Schematic diagram of tdTomato expression in PDGFRβ‐iCreERT2; tdTomato mice. (F) The mouse genotype was identified by tail DNA PCR and Southern blot analysis. Mouse genotype: 1, PDGFRβ‐iCreERT2+/+; 2, PDGFRβ‐iCreERT2+/−; 3, PDGFRβ‐iCreERT2−/−; 4, tdTomato+/+; 5, tdTomato+/−; 6, tdTomato−/−. (G) Expression of tdTomato in the superficial renal cortex in PDGFRβ‐iCreERT2; tdTomato mice with IRI. The data are presented as follows: error bars, mean ± SD; ns, not significant; *p < 0.05, ****p < 0.0001 versus Sham; Scale bar = 100 μm; IRI, renal ischaemia–reperfusion injury; uIRI, unilateral IRI model.

The uIRI model was successfully established by clamping the left kidney, and the functional contralateral kidney remained to ensure survival. To determine serum biochemical changes induced by IRI in the kidney, the contralateral kidney was removed 24 h before sampling. Blood and left kidney tissue were collected on the 1st, 3rd, 5th, 7th, 14th, 21st, and 28th days after reperfusion (Figure 1A).

During AKI‐CKD progression, the levels of Scr (Figure S1A) and BUN (Figure S1B) increased significantly from the 1st day of reperfusion and remained high until the 28th day, indicating the severity of kidney injury. Moreover, the IRI kidney weight/mouse body weight ratio increased from the 1st to 7th days of reperfusion but decreased from the 14th to 28th days of reperfusion (Figure S1C). Masson staining was performed to assess the area of fibrosis in the mouse kidney interstitial space (Figure 1B,C). The results showed that the degree of renal fibrosis was progressively exacerbated during AKI‐CKD.

As an important marker of renal pericytes, PDGFRβ was highly expressed in the renal interstitial space during the progression from AKI to CKD (Figure 1D). To trace the pericyte and their progeny, they were labelled with red fluorescence (Figure 1E). Mouse genotypes were determined by tail DNA PCR and Southern blot analysis (Figure 1F). The mice were surgically implanted with abdominal imaging windows on the left side of the abdomen for intravital observation via two‐photon microscopy (Figure S1D). High levels of red fluorescence in the renal interstitial space were detected (Figure 1G). On the 28th day of reperfusion, compared with low red fluorescence in the contralateral kidney, many PDGFRβ^+^ cells were observed around the tubules in the IRI kidney in the same mouse (Figure S1E), indicating that the PDGFRβ^+^ cell lineage proliferated and migrated to surround renal tubules during AKI‐CKD. The UUO mice also demonstrated elevated PDGFRβ expression in the renal interstitium (Figure S1F). Moreover, our previous study has provided evidence of PDGFRβ expression in the kidneys of human CKD patients.^14^

### 3.2 Renal pericytes transdifferentiated into myofibroblasts and persistently expressed PKM2 during AKI‐CKD

**FIGURE 2:**
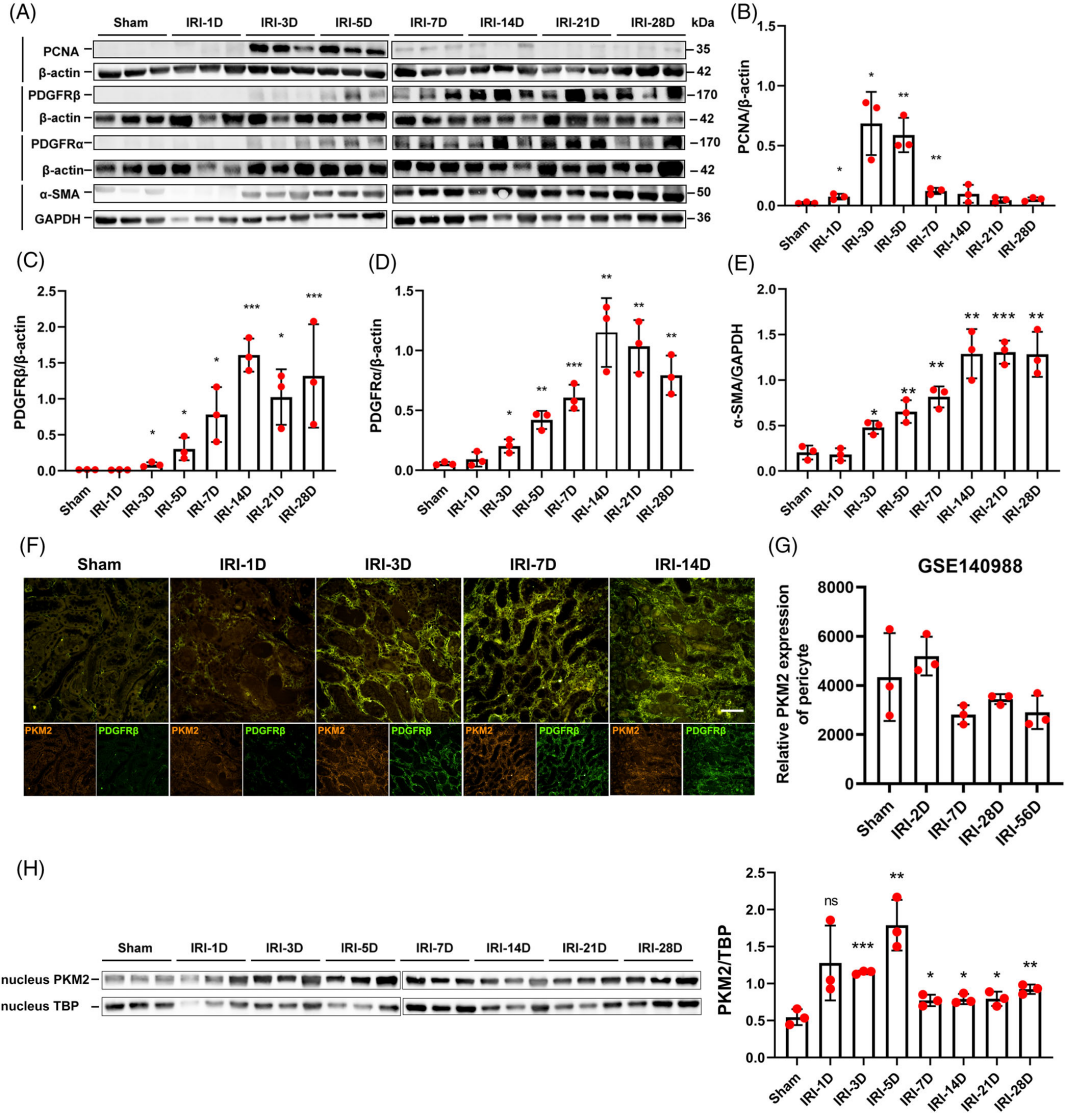
Pericytes continually expressed PKM2 during the progression of acute kidney injury‐chronic kidney disease. (A‐E) Expression of PCNA, PDGFRβ, PDGFRα, and α‐SMA in IRI kidney samples, as detected by Western blotting (*n* = 3). (F) The location of PDGFRβ and PKM2 expression in PDGFRβ‐iCreERT2; mTmG mice IRI kidney samples, as detected by immunofluorescence staining. (G) PKM2 mRNA was continually expressed in mouse renal pericytes after IRI (dataset GSE140988) (*n* = 3). (H) The expression of PKM2 in the nucleus of renal PDGFRβ‐positive pericytes sorted from IRI kidney samples, as detected by Western blotting (*n* = 3). The data are presented as follows: Error bars, mean ± SD; Scale bar = 100 μm; ns, not significant; *p < 0.05, **p < 0.01, ***p < 0.001 versus Sham. GAPDH, glyceraldehyde‐3‐phosphate dehydrogenase; IRI, renal ischaemia–reperfusion injury; PCNA, proliferative cell nuclear antigen; PDGFRβ, platelet‐derived growth factor receptor β; PKM2, pyruvate kinase M2; TBP, TATA binding protein.; α‐SMA, alpha smooth muscle actin.

To examine the fate of pericytes during AKI‐CKD, the expression levels of PCNA, PDGFRβ, PDGFRα, and α‐SMA in the kidney were detected by Western blotting (Figure 2A). The results showed that the expression of the proliferation‐related indicator PCNA peaked on the third to fifth days after surgery (Figure 2B). Consistent with the immunofluorescence staining results in Section 3.1, PDGFRβ began to increase on the 3rd day and was significantly upregulated from the 7th to 28th days of reperfusion (Figure 2C). Moreover, PDGFRα and α‐SMA, which are indicators of renal pericyte‐myofibroblast transdifferentiation, were also significantly upregulated during the chronic progression of kidney IRI (Figure 2D,E).

In the AKI‐CKD model, α‐SMA was expressed in activated renal pericytes and myofibroblasts, as well as in vascular smooth muscle cells. Immunofluorescence colocalization staining showed that PKM2 was expressed in PDGFRβ^+^ pericyte‐derived cells and α‐SMA^+^ renal interstitial cells at different time points during AKI‐CKD (Figures 2F and S2G). PKM2 was predominantly expressed in pericytes rather than tubular epithelial cells during the development of IRI‐induced CKD (Figures 2F and S1G). In the GEO dataset GSE140988,^15^ PKM2 mRNA was expressed in renal Col1a1^+^ pericytes in the IRI‐induced AKI‐CKD mouse model (Figure 2G), which is consistent with our results. Furthermore, Western blotting demonstrated an elevated expression of PKM2 in the nucleus of pericytes sorted from IRI kidney (Figure 2H).

### 3.3 Shikonin and TEPP‐46 alleviated renal pericyte‐myofibroblast transdifferentiation in vitro

**FIGURE 3:**
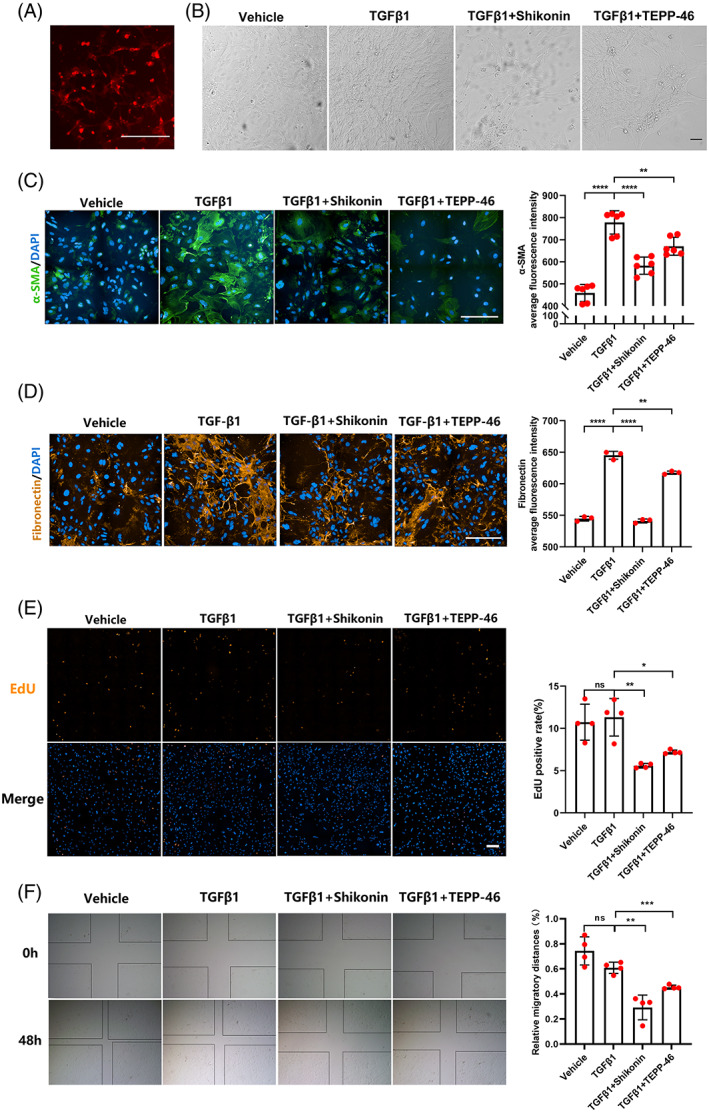
Shikonin and TEPP‐46 inhibited pericyte transdifferentiation, proliferation and migration in vitro. (A) Red fluorescence expression of primary renal pericytes extracted from PDGFRβ‐iCreERT2; tdTomato mice. (B) The morphology of renal pericytes as identified by a light microscope. (C,D) The expression levels of α‐SMA (C) and fibronectin (D) observed by immunofluorescent staining (*n* = 3–6). (E) Images showing EdU staining and EdU‐positive rates in renal pericytes (*n* = 4). (F) The migration of renal pericytes determined by scratch experiments (*n* = 4). The data were presented as follows: Error bars, mean ± SD; ns, not significant; *p < 0.05, **p < 0.01; ***p < 0.001, ****p < 0.0001; Scale bar = 200 μm. DAPI, 4,6‐diamidino‐2‐phenylindole; EdU, 5‐ethynyl‐2′‐deoxyuridine; FN, fibronectin; PDGFRβ, platelet growth factor receptor beta; PKM2, pyruvate kinase M2; TGFβ1, transforming growth factor β1; α‐SMA, alpha smooth muscle actin.

After tamoxifen administration, primary renal pericytes that were extracted from PDGFRβ‐iCreERT2; tdTomato mice expressed red fluorescence, indicating that renal pericytes could be purified by our sorting method (Figure 3A). Under a light microscope, the renal pericytes were small and mostly triangular. After TGFβ1 (5 ng/mL) treatment for 48 h, the cells were mostly polygonal or spindle shaped (Figure 3B). TGFβ1 induced α‐SMA and FN expression in pericytes, and these factors were downregulated by Shikonin and TEPP‐46 intervention, indicating that Shikonin and TEPP‐46 inhibited pericyte‐myofibroblast transdifferentiation (Figure 3C,D). Additionally, Shikonin and TEPP‐46 intervention inhibited the EdU‐positive rate of pericytes (Figure 3E). Moreover, the cell scratch test results showed that Shikonin and TEPP‐46 significantly inhibited pericyte migration (Figure 3F). Since Shikonin and TEPP‐46 attenuated renal pericyte‐myofibroblast transdifferentiation, we further investigated the mechanism by which PKM2 acts on pericyte transformation.

### 3.4 PKM2‐regulated renal pericyte‐myofibroblast transdifferentiation by transcriptionally inducing lactate production in vitro

**FIGURE 4:**
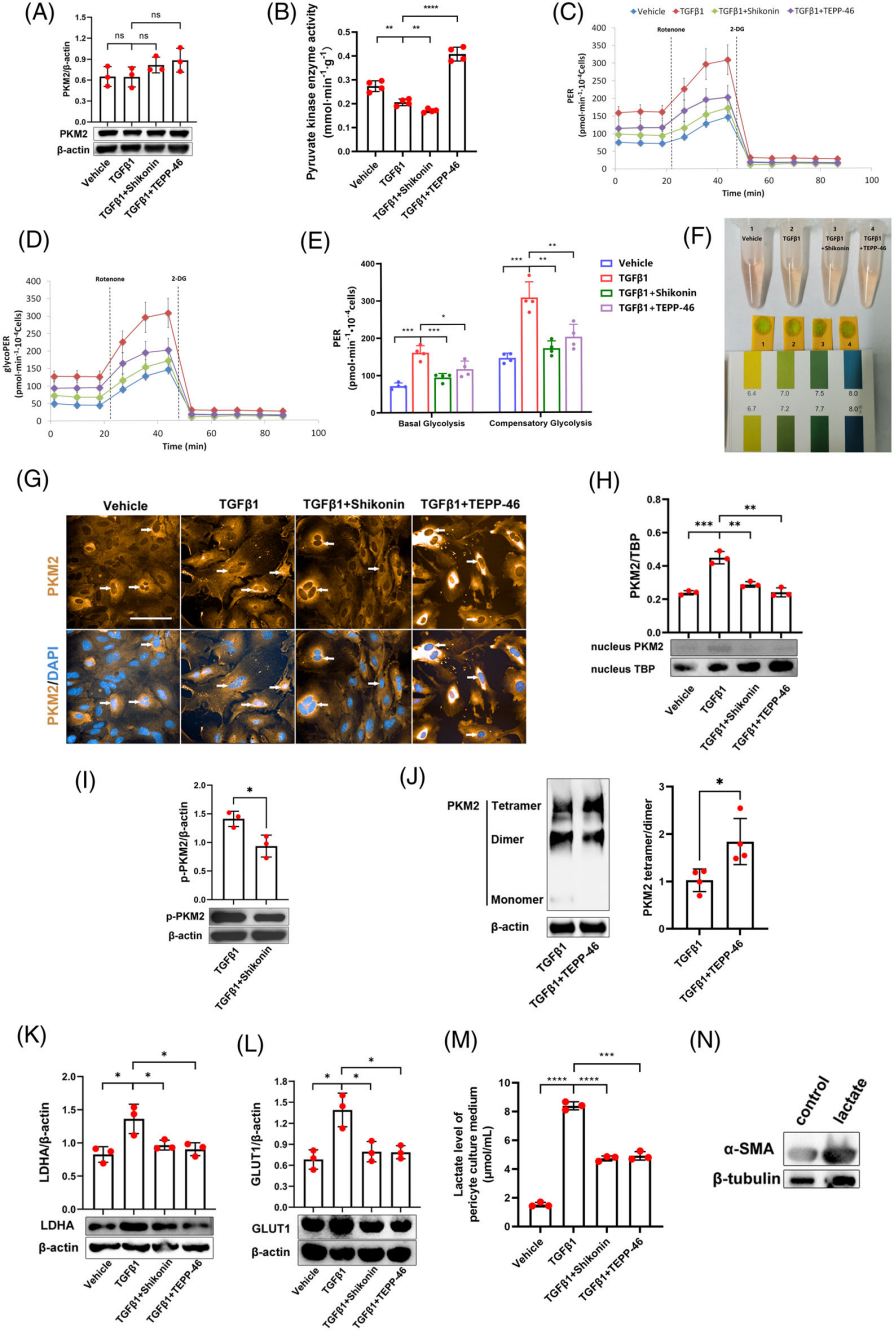
PKM2 influences renal pericyte‐myofibroblast transdifferentiation by transcription‐induced lactate production in vitro. (A) Expression of PKM2 in renal pericytes, as detected by Western blotting (*n* = 3). (B) Pyruvate kinase enzymatic activity in renal pericytes as investigated in vitro by pyruvate kinase enzyme activity assays (*n* = 4). (C,D) Total PER profile (C) and glycolytic PER profile (D) in renal pericytes, as detected by Seahorse experiments (*n* = 4). (E) Basal glycolysis PER and compensatory glycolysis PER of renal pericytes, as detected by Seahorse experiments (*n* = 4). (F) Photograph showing renal pericyte culture media and pH test strips. (G) The location of PKM2, as observed by immunofluorescent staining. White arrows indicate PKM2 in the nucleus of renal pericytes. (H) The expression of PKM2 in the nucleus of renal pericytes, as detected by Western blotting (*n* = 3). (I) The expression of p‐PKM2 Y105 in renal pericytes with or without Shikonin treatment, as detected by Western blotting (*n* = 3). (J) The expression of PKM2 in DSS crosslinked renal pericytes with or without TEPP‐46 treatment, as detected by Western blotting (*n* = 4). (K,L) The expression of LDHA (K) and GLUT1 (L) in renal pericytes, as detected by Western blotting (*n* = 3). (M) Lactate levels in pericyte culture media, as detected by a lactate assay kit (*n* = 3). (N) The expression of α‐SMA in renal pericytes with or without lactate treatment, as detected by Western blotting. The data are presented as follows: Error bars, mean ± SD; ns, not significant; *p < 0.05, **p < 0.01; ***p < 0.001, ****p < 0.0001; Scale bar = 100 μm. DAPI, 4,6‐diamidino‐2‐phenylindole; DSS, disuccinimidyl suberate; GLUT1, glucose transporter 1; glycoPER, glycolytic proton efflux rate; LDHA, lactate dehydrogenase A; PER, proton efflux rate; PKM2, pyruvate kinase M2; TBP, TATA binding protein; TGFβ1, transforming growth factor β1; α‐SMA, alpha smooth muscle actin.

We found that TGFβ1 did not significantly change the expression of PKM2 (Figure 4A) but decreased the enzymatic activity of PK (Figure 4B) in pericytes. However, in contrast with the effects on PK enzymatic activity, TGFβ1 significantly increased the glycolytic proton efflux rate (Figure 4C–E) of pericytes in the Seahorse experiment and acidified the culture medium (Figure 4F), indicating that TGFβ1 promoted glycolysis. We therefore hypothesized that PKM2 regulated renal pericyte‐myofibroblast transdifferentiation through nonglycolytic pathways.

PKM2 is also a transcriptional cofactor in tumours, and the translocation of PKM2 from the cytoplasm to the nucleus can regulate gene expression.^16^, ^17^ Immunofluorescence staining showed that nuclear expression of PKM2 increased after TGFβ1 intervention, while Shikonin and TEPP‐46 reduced nuclear PKM2 levels during pericyte transdifferentiation (Figure 4G). The Western blot results (Figure 4H) were consistent with the immunofluorescence staining results. Phosphorylation of PKM2 at Y105 alters its conformation and makes it easier to enter the nucleus.^16^ Shikonin blocked PKM2 phosphorylation at Y105 (Figure 4I), thereby inhibiting the nuclear entry of PKM2. The PKM2 subunit can exist as a tetramer, dimer, and monomer, and the dimer and monomer can enter the nucleus.^18^ Through DSS crosslinking and Western blotting, we demonstrated that TEPP‐46 could promote PKM2 tetramer formation, thus blocking the entry of PKM2 into the nucleus (Figure 4J). In the nucleus, PKM2 can promote the activation of genes such as LDHA and GLUT1 as a transcriptional cofactor.^17^, ^19^ Therefore, we further measured the expression of GLUT1 and LDHA in each group of pericytes. LDHA (Figure 4K) and GLUT1 (Figure 4L) expression in pericytes was upregulated by TGFβ1 and significantly downregulated by Shikonin or TEPP‐46. Lactate levels in the pericyte culture medium were significantly increased after TGFβ1 intervention and were decreased after Shikonin or TEPP‐46 intervention (Figure 4M). We further proved that lactate intervention promoted pericyte‐myofibroblast transdifferentiation by affecting the expression of α‐SMA (Figure 4N).

### 3.5 Shikonin and TEPP‐46 attenuated kidney fibrosis in AKI‐CKD in vivo

**FIGURE 5:**
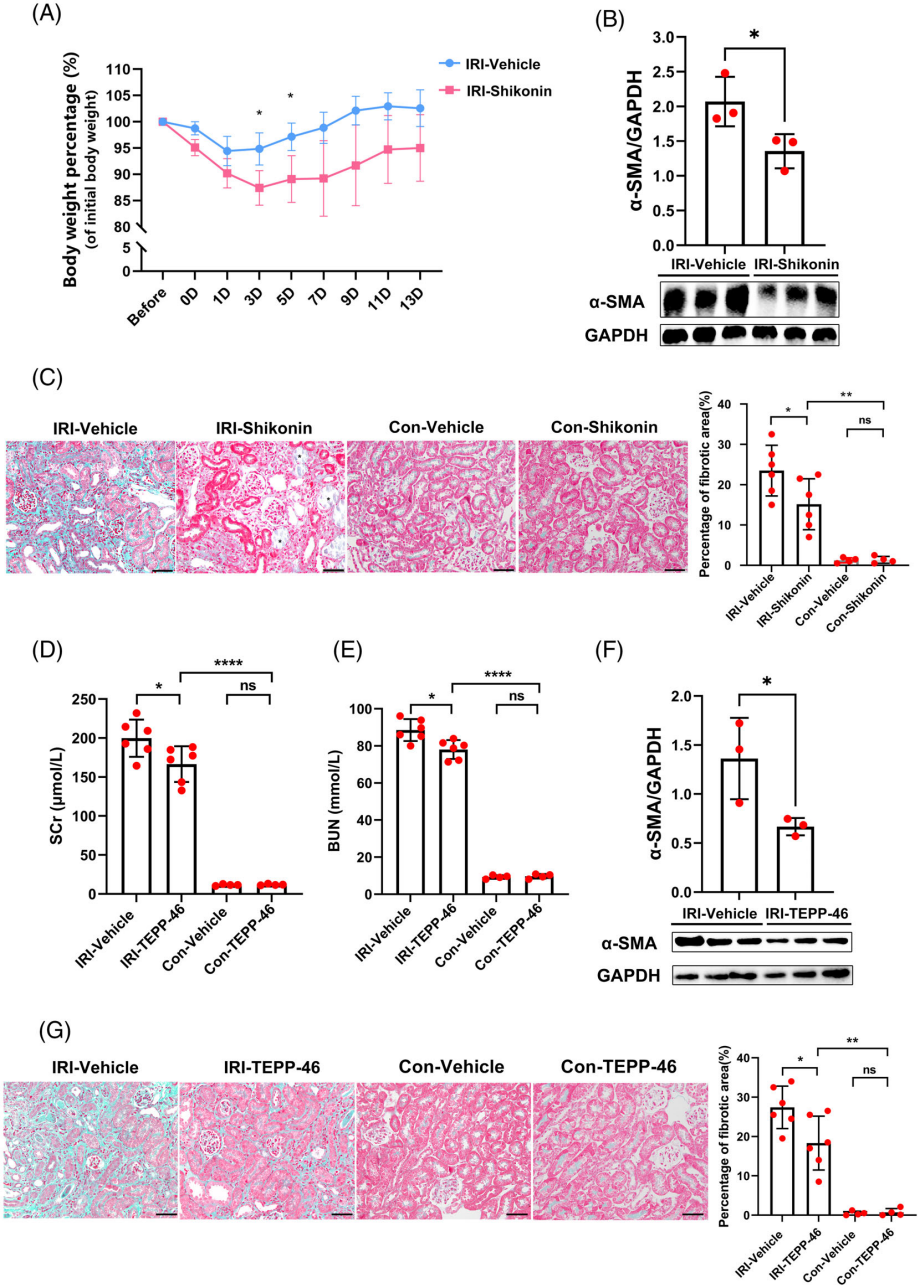
Shikonin and TEPP‐46 attenuated kidney fibrosis during acute kidney injury‐chronic kidney disease in vivo. (A) The mouse body weight percentage of the initial body weight in the IRI‐vehicle group and IRI‐Shikonin group (*n* = 6). (B) The expression of α‐SMA in mouse kidneys in the IRI‐vehicle group and IRI‐Shikonin group detected by Western blotting (*n* = 3). (C) Masson staining and the percentage of the fibrotic area in the kidney tissue of the IRI‐vehicle group, IRI‐Shikonin group, con‐vehicle group and con‐Shikonin group (*n* = 4–6). Asterisk, proteinaceous casts in the tubular lumen and tubular dilatation. (D) The levels of Scr in the IRI‐vehicle group, IRI‐TEPP‐46 group, con‐vehicle group and con‐TEPP‐46 group (*n* = 4–6). (E) The levels of BUN in the IRI‐vehicle group, IRI‐TEPP‐46 group, con‐vehicle group and con‐TEPP‐46 group (*n* = 4–6). (F) The expression of α‐SMA in the kidneys in the IRI‐vehicle group and IRI‐TEPP‐46 group detected by Western blotting (*n* = 3). (G) Masson staining and the percentage of the fibrotic area in kidney tissue in the IRI‐vehicle group, IRI‐TEPP‐46 group, con‐vehicle group and con‐TEPP‐46 group (*n* = 4–6). The data are presented as follows: Error bars, mean ± SD; ns, not significant; *p < 0.05, **p < 0.01; ****p < 0.0001; Bar = 50 μm. BUN, blood urea nitrogen; Contra, healthy contralateral kidney; IRI, renal ischaemia–reperfusion injury; Scr, serum creatinine; α‐SMA, alpha smooth muscle actin.

Since the expression of fibrotic pericytes peaked at 14 days (as shown in Section 3.2), the 14th day after surgery was used to investigate the effects of Shikonin and TEPP‐46 on AKI‐CKD mice. First, we administered Shikonin to IRI mice. We found that Shikonin intervention reduced body weight (Figure 5A; *p* < 0.001 by repeated measures analysis of variance [ANOVA]). Shikonin intervention significantly attenuated kidney α‐SMA expression in AKI‐CKD (Figure 5B) and renal tissue fibrosis (Figure 5C). Moreover, we observed proteinaceous casts in the tubular lumen and tubular dilatation in the Shikonin group (Figure 5C). Next, we treated IRI mice with TEPP‐46. Compared with vehicle treatment, TEPP‐46 decreased mice Scr (Figure 5D) and BUN (Figure 5E) levels. Similar to the effects of Shikonin, TEPP‐46 attenuated kidney α‐SMA expression during AKI‐CKD (Figure 5F) and ameliorated renal tissue fibrosis (Figure 5G). Shikonin and TEPP‐46 attenuated kidney fibrosis during AKI‐CKD.

## 4 DISCUSSION

The incidence of AKI is increasing yearly, and there are more than 10 million cases worldwide each year.^1^ As a prognostic indicator of AKI, kidney fibrosis affects millions of people worldwide.^20^ Renal pericytes play an important role in the progression of kidney fibrosis.^3^ In the healthy kidney, renal pericytes interact with vascular endothelial cells to regulate blood flow. When the kidney is damaged, pericytes are activated and migrate from capillaries to the renal interstitial space, promoting the repair of renal tubules.^21^ Furthermore, pericytes transform into myofibroblasts, which secrete collagen and other extracellular matrix components.^22^ Lineage tracing studies have shown that pericytes and fibroblasts are the main sources of myofibroblasts.

The function of PKM2 in multiple kidney diseases has received attention. Increased expression of PKM2 in fibrotic kidneys has been observed in animal models of renal IRI,^8^ UUO,^23^, ^24^ and diabetic kidney disease.^25^ Cheon et al^9^ found that PKM2 in urine was an early and sensitive biomarker of nephrotoxicity in AKI. Furthermore, on the fourteenth day after renal IRI, there was high PKM2 expression in renal proximal tubules and the interstitial space.^8^ Previous studies have shown that Shikonin attenuates UUO‐induced renal fibrosis in mice,^23^, ^24^ and TEPP‐46 inhibits the progression of diabetic renal fibrosis by inhibiting the epithelial–mesenchymal transition in renal podocytes.^25^ However, the use of Shikonin and TEPP‐46 in the AKI‐CKD model and studies of their effects on AKI‐CKD renal fibrosis are lacking.

PKM2 plays a role in fibrosis in various cells.^16^ Hepatic stellate cells are equivalent to hepatic pericytes, and PKM2 protects against hepatic stellate cell activation and hepatic fibrosis.^26^ Gao et al.^27^ found that PKM2 deletion markedly alleviated pulmonary fibroblast fibrosis. Ye et al.^28^ found that fibroblast‐specific knockout of PKM2 aggravated renal tubular damage in IRI or folic acid‐induced AKI.^28^ However, whether PKM2 is expressed in renal pericytes and whether it is involved in the fate of pericytes during AKI‐CKD require further investigation.

As a hyperperfused organ, the kidney is very sensitive to ischaemia–reperfusion, which is a common cause of clinical AKI.^29^ Animal models of AKI‐CKD caused by ischaemia–reperfusion include uIRI, uIRI with contralateral nephrectomy (uIRIx), and bilateral IRI (bIRI) models.^30^, ^31^ When kidney damage is mild, adaptive recovery from AKI occurs without the progression of kidney fibrosis.^30^ If the damage is severe, the kidney may not be fully repaired and may develop fibrosis.^32^ However, when the injury is too severe, the mortality rate of animals with IRI is high. With normal contralateral renal function, uIRI model animals have low mortality rates and can be observed for a long time. Compared with bIRI or uIRIx models, uIRI reduces renal perfusion, resulting in more severe tubular injury and increased susceptibility to the progression of renal fibrosis.^30^ In the uIRI model, renal creatinine and BUN levels remained high during AKI‐CKD, and renal interstitial fibrosis occurred during the chronic phase.

Renal pericytes originate from the mesoderm, are the supporting cells in the vascular endothelium, and are located on the non‐luminal side of the microvascular endothelium.^33^ Under normal physiological conditions, these cells are involved in the maintenance of microcirculation stability and the production of erythropoietin.^33^ Pericytes are often identified by genetic lineage tracing and specific markers. Common markers of renal pericytes include PDGFRβ, chondroitin sulphate proteoglycan (NG2), and CD73.^4^, ^34^ Other markers, such as zinc finger transcription factor (Gli1), have also been used to identify pericyte subpopulations.^35^ Different markers are often expressed by renal pericytes. For example, 60% of CD73^+^ cells co‐localized with PDGFRβ^+^ pericytes, and 15% of CD73^+^/PDGFRβ^+^ renal pericytes also expressed Gli1.^36^ Among them, PDGFRβ is a widely expressed pericyte marker that is often used in pericyte research.^37^, ^38^ We found that PDGFRβ was significantly expressed after ischaemia–reperfusion in uIRI models. Moreover, as markers of pericyte‐myofibroblast transdifferentiation, α‐SMA, and PDGFRα were significantly upregulated after surgery. We used a previously described method^39^ to purify primary renal pericytes. TGFβ is expressed during AKI‐CKD, is the main factor driving renal fibrosis, and is also a classic signal of pericyte transdifferentiation.^37^, ^40^ In vitro, TGFβ1 stimulation for 48 h successfully induced pericyte‐myofibroblast transdifferentiation.

PDGFRβ‐iCreERT2; tdTomato mice were injected with tamoxifen and underwent a 3‐week tamoxifen washout period before uIRI surgery. Since tamoxifen was absent in postoperative mice, the cells expressing red fluorescent protein in the experiment were all derived from PDGFRβ‐positive cells that were present before surgery. Our results demonstrated an increase in tdTomato‐expressing cells in the renal interstitium, as shown by two‐photon microscopy in vivo, which suggests massive renal pericytes proliferation during AKI‐CKD progression.

Proliferative cells express high levels of PKM2, which is a specific marker of embryos, tumours, and damaged organs.^16^ PKM2 has a weak glycolytic enzymatic activity similar to PK and can act as a transcriptional promoter that is essential for metabolic reprogramming.^41^ PK is a key enzyme in glycolysis that catalyses the conversion of phosphoenolpyruvate to pyruvate and promotes lactate production in the cytoplasm.^16^ As a transcriptional cofactor, PKM2 can enter the nucleus to regulate gene expression and promote the transcription of metabolism‐ and signalling pathway‐related molecules by interacting with various transcription factors.^16^ Shikonin can inhibit not only the glycolytic enzymatic activity of PKM2 but also its ability to promote transcription.^42^ TEPP‐46 promotes the glycolytic enzymatic activity of PKM2 and inhibits its transcriptional activity.^42^ Our results suggest that during TEPP‐46‐mediated regulation of glycolysis, the transcriptional effect of PKM2 is stronger than its enzymatic activity. Phosphorylation of p‐PKM2 at the Y105 position can stabilize the conformation of PKM2 in the nucleus.^43^, ^44^ Therefore, Shikonin inhibits the levels of p‐PKM2 Y105 and nuclear entry of PKM2, where it can act as a transcriptional cofactor. In our study, we indirectly proved the transcriptional regulation of PKM2 by detecting its nuclear translocation. This is supported by previous studies that have proved the transcriptional activity of PKM2.^16^, ^17^

Metabolic reprogramming, which is a concept that was first proposed by Warburg, refers to the tendency of cancer cells to undergo glycolysis in the cytoplasm even in an oxygen‐rich environment.^45^, ^46^ Recent studies have shown that metabolic reprogramming is closely related to pericyte differentiation. Nwadozi et al.^47^ found that the metabolic level of pericytes was low during the resting state but increased when pericytes proliferated and transdifferentiated. We demonstrated that TGFβ1 promoted pericyte PKM2 entry into the nucleus, upregulated LDHA and expression, and promoted lactate production, thereby regulating pericyte metabolic reprogramming. GLUT1 mediates cellular glucose uptake, while LDHA converts intracellular pyruvate to lactate, which promotes pericyte glycolysis and lactate formation. Lactate‐induced renal fibroblast activation has been described previously,^48^ and we proved that lactate‐induced pericyte‐myofibroblast transdifferentiation.

There are limitations to this study. An in vivo animal model of pericyte‐specific PKM2 deletion has not been established. In the next step in our research, we will construct PDGFRβ‐iCreERT2; Pkm2^fl/fl^ mice to compare renal injury and fibrosis during AKI‐CKD.

## 5 CONCLUSION

Our study demonstrated that PKM2 regulated the transdifferentiation of renal pericytes into myofibroblasts by regulating the transcriptional levels of LDHA and GLUT1, as well as lactate production. Therefore, reducing the nuclear translocation of PKM2 may inhibit the transdifferentiation of renal pericytes to myofibroblasts. Moreover, blocking the entry of PKM2 into the nucleus may inhibit excessive lactate‐induced renal interstitial fibrosis, which may provide new ideas for the treatment of AKI‐CKD.

## AUTHOR CONTRIBUTIONS

Xiangmei Chen, Lingling Wu, and Xueyuan Bai designed the study. Yulan Chen, Jianwen Chen, Mengjie Huang, Yan Zhang, Jiaona Liu, and Xu Wang performed the experiments. Quan Hong, Qing Ouyang, and Xuefeng Sun optimized the research. Yulan Chen drafted the article. Xiangmei Chen, Lingling Wu, and Xueyuan Bai supervised the study and edited the article. All authors contributed to the article and approved the submitted version.

## FUNDING INFORMATION

This work was supported by the National Natural Science Foundation of China (Nos. 82030025, 82100713, 81830060 and 32200579).

## CONFLICT OF INTEREST STATEMENT

All authors confirm that there are no conflicts of interest.

## Supporting information


**FIGURE S1:** (A) The levels of Scr at different time points (*n* = 6). (B) The levels of BUN at different time points (*n* = 6). (C) IRI kidney weight as a percentage of mouse body weight (*n* = 6). (D) Mouse abdominal imaging window for kidney microscopy. (E) Comparison of tdTomato expression between IRI kidneys and contralateral kidneys in the same PDGFRβ‐iCreERT2; tdTomato mouse at 28 days. (F) Location of PDGFRβ in mouse UUO kidney samples, as investigated by immunohistochemical staining. (G) The location of α‐SMA and PKM2 expression in mouse IRI kidney samples, as detected by immunofluorescence staining. The data are presented as follows: error bars, mean ± SD; *p < 0.05, **p < 0.01, ***p < 0.001, ****p < 0.0001 versus Sham. BUN, blood urea nitrogen; Contra, healthy contralateral kidney; IRI, renal ischaemia–reperfusion injury; Scr, serum creatinine; UUO, unilateral ureteral obstruction.

## Data Availability

All data and models generated and used during the study are available from the corresponding author upon reasonable request.
