# Supplementary figures and images for: Pyruvate kinase M2 regulates kidney fibrosis through pericyte glycolysis during the progression from acute kidney injury to chronic kidney disease
Source: Cell Prolif. 2023 Sep 25;57(2):e13548. doi: 10.1111/cpr.13548 (PMC10849781; doi:10.1111/cpr.13548)

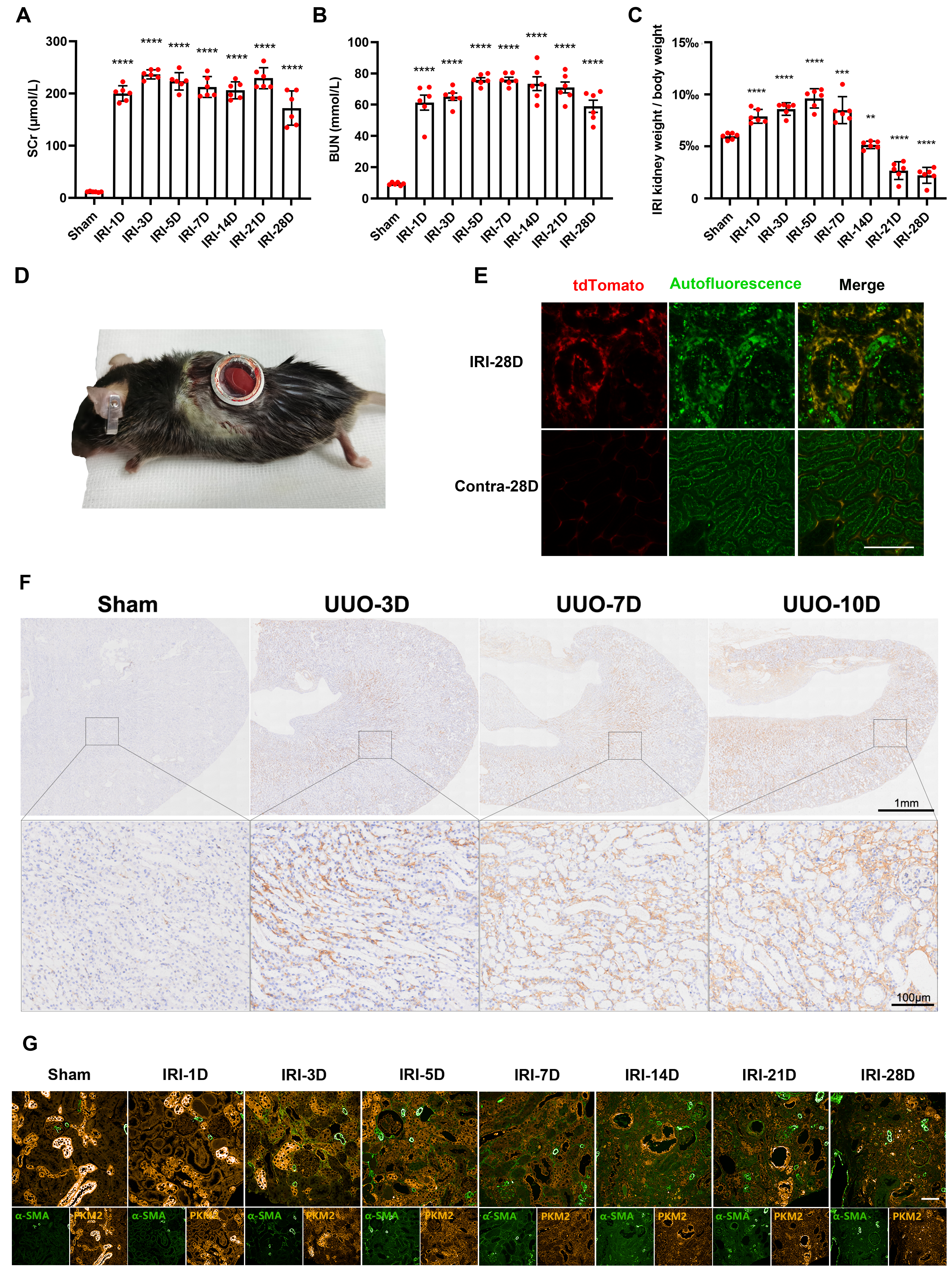

Supplement: Supplementary file 1 — FIGURE S1: (A) The levels of Scr at different time points (n = 6). (B) The levels of BUN at different time points (n = 6). (C) IRI kidney weight as a percentage of mouse body weight (n = 6). (D) Mouse abdominal imaging window for kidney microscopy. (E) Comparison of tdTomato expression between IRI kidneys and contralateral kidneys in the same PDGFRβ‐iCreERT2; tdTomato mouse at 28 days. (F) Location of PDGFRβ in mouse UUO kidney samples, as investigated by immunohistochemical staining. (G) The location of α‐SMA and PKM2 expression in mouse IRI kidney samples, as detected by immunofluorescence staining. The data are presented as follows: error bars, mean ± SD; *p < 0.05, **p < 0.01, ***p < 0.001, ****p < 0.0001 versus Sham. BUN, blood urea nitrogen; Contra, healthy contralateral kidney; IRI, renal ischaemia–reperfusion injury; Scr, serum creatinine; UUO, unilateral ureteral obstruction. [file CPR-57-e13548-s001.tif]
